# Beliefs and Opinions of Health Care Workers and Students Regarding Influenza and Influenza Vaccination in Tuscany, Central Italy

**DOI:** 10.3390/vaccines3010137

**Published:** 2015-02-26

**Authors:** Guglielmo Bonaccorsi, Francesca Santomauro, Barbara Rita Porchia, Giuditta Niccolai, Elettra Pellegrino, Paolo Bonanni, Chiara Lorini

**Affiliations:** 1 Department of Clinical and Experimental Medicine, University of Florence, viale GB Morgagni 48, 50134 Florence, Italy; E-Mail: guglielmo.bonaccorsi@unifi.it; 2 Department of Health Science, University of Florence, viale GB Morgagni 48, 50134 Florence, Italy; E-Mails: francesca.santomauro@unifi.it (F.S.); paolo.bonanni@unifi.it (P.B.); 3 School of Specialization in Hygiene and Preventive Medicine, University of Florence, viale GB Morgagni 48, 50134 Florence, Italy; E-Mails: babibell83@gmail.com (B.R.P.); giuditta.niccolai@sysoft.it (G.N.); elettra.pellegrino@gmail.com (E.P.)

**Keywords:** attitudes, knowledge, influenza, health care workers, health professions students

## Abstract

Immunization of health care workers (HCWs) against influenza has been associated with improvements in patient safety. The aim of this study is to assess the beliefs, attitudes, and knowledge of HCWs and health profession students regarding influenza. An anonymous questionnaire was distributed to HCWs in three local Florentine healthcare units, at Careggi University Teaching Hospital, and to students in health profession degree programs. A total of 2576 questionnaires were fully completed. A total of 12.3% of subjects responded that they were “always vaccinated” in all three of the seasonal vaccination campaigns studied (2007–2008 to 2009–2010), 13.1% had been vaccinated once or twice, and 74.6% had not received vaccinations. Although the enrolled subjects tended to respond that they were “never vaccinated,” they considered influenza to be a serious illness and believed that the influenza vaccine is effective. The subjects who refused vaccination more frequently believed that the vaccine could cause influenza and that it could have serious side effects. More than 60% of the “always vaccinated” group completely agreed that HCWs should be vaccinated. Self-protection and protecting family members or other people close to the respondent from being infected and representing potential sources of influenza infection can be considered motivating factors for vaccination. The results highlight the importance of improving vaccination rates among all HCWs through multi-component interventions. Knowledge of influenza should be reinforced.

## 1. Introduction

Influenza is a major public health challenge. The most effective way to prevent infection with the influenza virus and its potentially severe complications is immunization. Vaccination is safe and well-tolerated and can prevent up to 70%–90% of influenza cases in healthy adults [1,2].

Health care workers (HCWs) and medical students not only are at risk of exposure to influenza viruses because of their contact with patients but are also considered a potential source of transmission. The World Health Organization and the United States Centers for Disease Control and Prevention recommend influenza vaccination for health care personnel, health profession students, and trainees [1,3] to protect themselves and their patients.

Immunization of HCWs has been associated with improvements in patient safety and decreased severe morbidity and mortality in hospitals and other health care facilities [4,5,6,7,8,9]. Moreover, vaccination of HCWs can reduce workplace absences, deliver economic benefits for healthcare systems, and provide cost savings for healthcare organizations [10,11].

Assessing the beliefs, attitudes, and knowledge of HCWs and health profession students regarding influenza and the influenza vaccine can be useful for planning tailored influenza vaccination campaigns and improving vaccination rates [12,13].

## 2. Materials and Methods

### 2.1. Setting

As described in other publications [14,15], the survey was conducted between October 2010 and April 2011 in the Florence metropolitan area, and the data were collected between October and November 2010. In Italy, the seasonal influenza vaccination is offered free of charge every year to all HCWs and students, and the same has happened for the pandemic influenza vaccination.

We recruited the study sample from the employees of the Local Health Units (LHUs) of Empoli and Pistoia, at Careggi University Teaching Hospital, and among students in the final two years of degree programs in Medicine, Nursing, Health Care Assistance, and Techniques of Prevention in the Environment and in the Workplace in Florence.

In the LHUs of Empoli and Pistoia, and in the Careggi University Teaching Hospital, the total number of employees, including medical, paramedical, and non-medical workers, was 11,369, overall, while the students of the final two years of degree programs included in the survey totaled approximately 920.

The study was conducted according to the Helsinki Declaration of 1975.

### 2.2. Questionnaire and Sampling Mode

The tool used for the survey was an anonymous self-administered questionnaire with closed questions. The questionnaire was formulated and shared in a peer group, then validated in a survey of 95 HCWs.

The questionnaire was composed of three sections. In the first section, the study participants recorded their agreement or disagreement (“totally agree,” “partially agree,” “partially disagree,” or “totally disagree”) with factual statements regarding influenza and the influenza vaccine.

In the second section, the participants indicated their previous influenza vaccination status (against pandemic influenza in 2009 and against seasonal influenza between 2007 and 2009) and their reasons for having been vaccinated or not vaccinated. The participants were also asked about their role in administering the influenza vaccine, their preferred type of influenza vaccine, and which categories of patients they would recommend receive vaccinations.

The third section included questions on the participants’ socio-demographic characteristics, their degree qualifications, home department, whether they lived with people at risk to transmit influenza (children under 9 years) or to get flu (people over 65 years and/or with chronic illness), and their history of chronic cardiovascular disease, chronic respiratory disease, chronic renal failure, diabetes, autoimmune diseases, and respiratory infections in the previous years. Finally, the questionnaire included an assessment of self-perceived health status (from 1 for “bad” to 10 for “excellent”) that was subsequently grouped into two categories (“not good” from 1 to 5 and “good” from 6 to 10).

The sampling criteria are explained in previous publications [14,15]. The study was conducted on a convenience sample. We recruited the subjects among medical and paramedical employees; for the Careggi University Teaching Hospital we limited the survey to the Emergency Department as well as the Departments of Obstetrics and Gynecology, Cardiology, and General Surgery. A small number of non-HCWs working in the LHUs under examination was also included (administrative staff and cleaning company employees), to compare attitudes and beliefs.

Recruitment was performed according to different approaches: the study team asked HCWs to complete the questionnaire and to return it to a locked box located within the department, or directly administered the questionnaire during mandatory training events. The students were recruited during their classes.

The total number of questionnaires distributed was 3597, of which 2627 (73%) were returned.

### 2.3. Statistical Analysis

In this paper, we analyze the first and the third sections of the questionnaire, focusing on perceptions of the risk of contracting and transmitting influenza, attitudes regarding being vaccinated, and knowledge of vaccine effectiveness and safety. The other sections have been discussed in other papers, as well as predictors of vaccination against pandemic influenza [14,15].

The collected data were entered into an *ad hoc* database. The statistical analysis was performed using IBM SPSS Statistics 20 (SPSS Inc., Chicago, IL, USA, 2011).

Data regarding previous vaccination against seasonal influenza were re-coded into three categories: “always vaccinated” (in 2007, 2008, and 2009); “sometimes vaccinated” (once or twice from 2007 to 2009); and “never vaccinated” (not vaccinated in 2007, 2008, or 2009).

A descriptive analysis was performed, including an evaluation of associations using the Chi^2^ test. An alpha level of 0.05 was considered significant.

## 3. Results

### 3.1. Descriptive Analysis

Of a total of 2627 questionnaires, 2576 were fully completed. Three hundred seventeen subjects answered “always vaccinated,” which indicated they had been vaccinated in all three seasonal vaccination campaigns under consideration (2007–2008 to 2009–2010), and 338 reported receiving vaccinations once or twice. In contrast, 1921 subjects had never been vaccinated during the campaigns under consideration. The data are displayed in Table 1. During the three years under examination, the vaccination rates showed no significant changes (18.3% in 2007, 17.8% in 2008, and 18.1% in 2009).

The analysis of the results by gender indicates that vaccination continuity (meaning subjects were vaccinated in 2007–2008, 2008–2009, and 2009–2010) was more common in males than in females (18.3% *vs.* 9.8%).

As educational levels increase, there is a greater tendency to be “always vaccinated” rather than “sometimes vaccinated.” One exception to this is the category of high school graduates, where this trend is not confirmed.

Among all occupational categories, physicians have the lowest proportion of “never vaccinated” (48.4%), followed by non-healthcare workers (administrative staff and cleaning company employees).

Nurses, healthcare assistants, and other HCWS have a high percentage of “never vaccinated” (71.9%–81.4%). This is also the case for students (79.7%). The highest proportion of “always vaccinated” (35.3%) is found among physicians, followed by non-healthcare workers.

The subjects living with elderly people or with people affected by chronic illnesses had a greater tendency to receive vaccinations in all three seasons than to receive them once or twice in the three seasonal vaccination campaigns studied. This is also observed in the subjects affected by chronic illnesses such as cardiovascular, respiratory, and autoimmune diseases, kidney-related diseases, and diabetes.

Living with children younger than nine years of age or having had a respiratory infection in recent years does not influence vaccination continuity.

Those who believe that their own health is “not good” are observed in greater numbers in the “always vaccinated” subgroup than the “vaccinated once or twice” subgroup.

Furthermore, the “always vaccinated” subjects have a higher mean age than the other subgroups.

### 3.2. Knowledge about Influenza, Vaccines, and Vaccination

**Figure 1:**
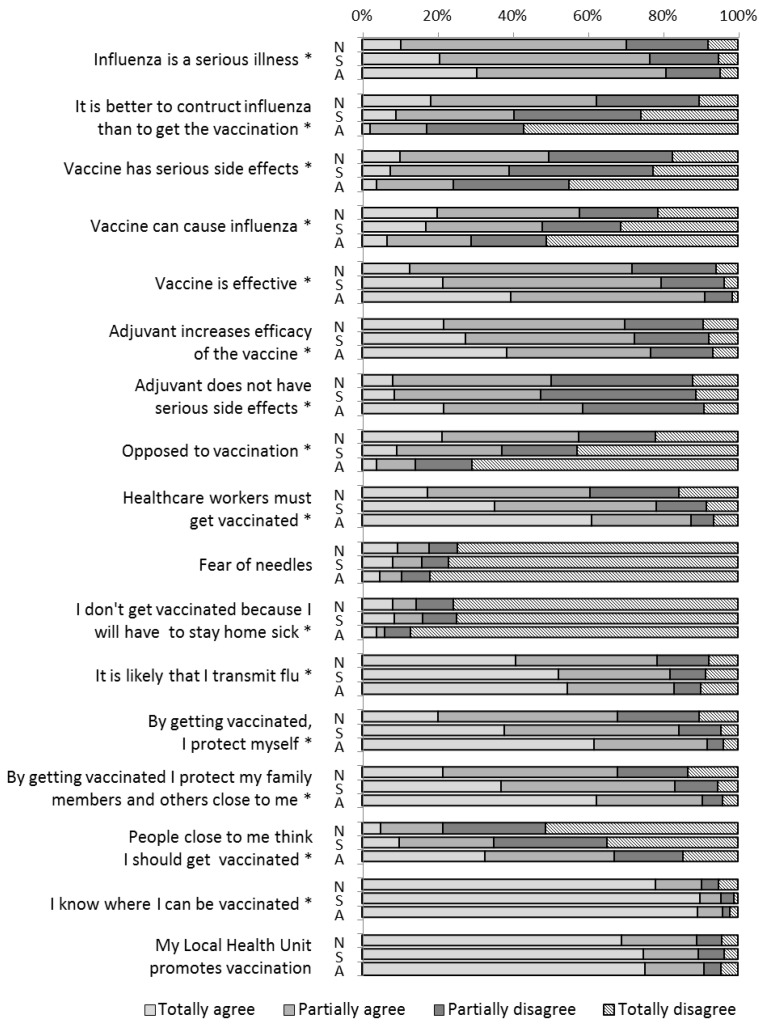
Agreement or disagreement with factual statements regarding influenza, influenza vaccination, and vaccines in the “never vaccinated” (N), “sometimes vaccinated” (S), and “always vaccinated” (A) subgroups. * *p* < 0.05.

Figure 1 illustrates the participants’ agreement or disagreement with factual statements regarding influenza, influenza vaccination, and vaccines, and attitudes towards them. For each statement, the data were analyzed to compare the previously identified three categories according to vaccination status.

**Table 1:** Seasonal influenza vaccination coverage from 2007–2008 to 2009–2010.

Variables	Total (N = 2576)		Coverage % Never Vaccinated	Coverage % Vaccinated Once or Twice	Coverage % Always Vaccinated
	N	% ^	(N = 1921; 74.6%) °	(N = 338; 13.1%) °	(N = 317; 12.3%) °
Gender					
Male	660	*25.6*	66.5	15.2	18.3
Female	1809	*70.2*	77.8	12.4	9.8
Educational qualification					
Elementary school	7	*0.3*	71.4	14.3	14.3
Middle school	167	*6.5*	77.2	9.6	13.2
High school	1444	*56*	79.2	12.2	8.7
University degree	624	*24.2*	70.4	14.7	14.9
Postgraduate	208	*8.1*	52.4	18.3	29.3
Occupational category					
Physicians	258	*10*	48.4	16.3	35.3
Nurses	1010	*39.2*	78.8	12.1	9.1
Health care assistants	102	*3.9*	81.4	6.9	11.8
Other healthcare workers	391	*15.2*	71.9	14.6	13.6
Non-healthcare workers	97	*3.8*	69.1	8.2	22.7
Students	601	*23.3*	79.7	14.6	5.7
Children <9 years in household					
Yes	578	*22.4*	73.4	15.2	11.4
People >65 years in household					
Yes	513	*19.9*	69.8	13.8	16.4
People with chronic illness in household					
Yes	324	*12.6*	66	15.7	18.2
Chronic Cardiovascular disease					
Yes	34	*1.3*	38.2	20.6	41.2
Chronic Respiratory Disease					
Yes	149	*5.8*	59.7	10.7	29.5
Chronic Kidney-related Disease					
Yes	14	*0.5*	64.3	14.3	21.4
Diabetes					
Yes	34	*1.3*	41.2	14.7	44.1
Autoimmune Disease					
Yes	109	*4.2*	67.9	15.6	16.5
Respiratory infection in the previous year					
Yes	288	*11.2*	68.4	19.1	12.5
Perceived health status					
Not good (1–5)	135	*5.2*	60.7	17	22.2
Good (6–10)	2441	*94.8*	75.3	12.9	11.8
Age^+^					
N (%)	2365 (91.8)		1768 (68.6)	308 (11.9)	289 (11.2)
Mean (DS)	38.3 (11.6)		37.5 (11.3)	37.4 (11.5)	44.4 (11.1)
Range	18–66		19–65	20–66	18–65

^ The difference between 100% and the sum of the percentages of each variable corresponds to missing values; ° For all categorical variables, distribution compared to seasonal influenza vaccination (never vaccinated, vaccinated once or twice, always vaccinated): *p* < 0.05 (Chi^2^ test); ^+^ Comparison between averages for seasonal influenza for those never vaccinated, vaccinated once or twice, or always vaccinated: *p* < 0.05 (ANOVA).

The three categories were significantly associated with each statement, with the exception of “fear of needles” and “my Local Health Unit promotes vaccination.”

Compared to those who responded that they were “never vaccinated” or “sometimes vaccinated,” those who responded “always vaccinated” were more likely to believe that influenza is a serious illness.

Moreover, 54.7% of those “always vaccinated,” 52.3% of those “sometimes vaccinated,” and 40.9% of those “never vaccinated” totally agree with the statement “it is likely that I can transmit influenza.”

Among the “never vaccinated” subjects, 10.4% totally disagree that it is better to contract influenza than to get vaccinated; the percentage of disagreement increases in the “sometimes vaccinated” and “always vaccinated” subgroups.

Regarding the effectiveness of the influenza vaccine, the percentage of agreement (considering both total and partial agreement) is high for each category (greater than 71.7%).

The subjects who refused vaccination have a greater tendency to believe that the vaccine could cause influenza and that it could have serious side effects than those in the other two subgroups.

Agreement regarding adjuvant effectiveness increases from those who were never vaccinated to those who were always vaccinated; in contrast, there is no significant difference between the three categories in beliefs regarding possible side effects caused by adjuvants.

Most of the respondents know where the vaccine is offered and are aware that the Local Health Unit promotes vaccination.

### 3.3. Attitudes towards Vaccination

The percentage of subjects opposed to vaccination (those in partial and total agreement with the statement) is 57.7% in the “never vaccinated” group, 37.3% in the “sometimes vaccinated” group, and 14.2% in the “always vaccinated” group.

More than 60% of the subjects in the “always vaccinated” group totally agreed that HCWs should be vaccinated, but only 17.5% of the “never vaccinated” group shared that opinion.

Self-protection and protecting family members and other people close to the HCWs from being infected and representing potential sources of influenza can be considered one of the motivating factors for subjects to receive the vaccine.

The percentage of subjects who were “always vaccinated” is higher than those who were “sometimes vaccinated” or “never vaccinated” (32.6%, 9.9%, and 4.9%, respectively) among the respondents who believed that the flu vaccine is important to their family members or other people close to them.

## 4. Discussion

The results of the survey show that the enrolled subjects tend to be in the “never vaccinated” category despite considering influenza to be a serious illness and believing that the influenza vaccine is effective.

Because a person’s history of influenza vaccination is considered a positive predictor of future vaccination [14,15,16], we chose to focus on that particular aspect: the continuity of vaccination over time, in accordance with other studies [17,18]. Like these authors, we found that influenza vaccination continuity was more common in males than females, and among people with a higher mean age; moreover, a major chronic condition increases compliance with vaccination.

We paid attention to attitudes and motivators driving vaccine acceptance. These include protection of oneself and family members or other close people, and the belief that influenza is a serious illness and that the vaccine against it is effective. Past research supports our findings [16,19,20,21].

Awareness that influenza is a serious illness with an effective vaccine and understanding that one may transmit influenza are all lower in the “never vaccinated” group than in the “always vaccinated” and “sometimes vaccinated” groups. For this reason, it is important to increase the level of knowledge regarding the risk of acquiring and transmitting influenza in the workplace and to improve the adoption of preventive measures, namely, immunization in this group. For the “always vaccinated” and “sometimes vaccinated” groups, the purpose of public health interventions could be to maintain and support their level of influenza awareness and knowledge through informational campaigns in workplaces.

As has been previously demonstrated [22,23], physicians exhibit a stronger tendency to receive vaccines. This finding supports the need for professional-sensitive programs to increase vaccination coverage, taking into account different levels of knowledge and attitudes towards influenza and influenza vaccination among various occupational categories [22].

The survey also investigated possible barriers to vaccination. Contrary to findings in other publications [24,25], in this research, organizational issues are not perceived to be barriers: in fact, subjects know where they can be vaccinated and they are aware that their Local Health Unit promotes vaccination. Regarding attitudes against vaccination, it is noteworthy that the HCWs who were not vaccinated in the three seasons considered do not perceive immunization against influenza to be a duty of a health professional. Moreover, a relatively small proportion of HCWs are still opposed to vaccination in general, so it would be interesting to understand their reasons for maintaining this position.

## 5. Conclusions

In Italy, other studies have been conducted to investigate the attitudes and knowledge of HCWs and medical students regarding influenza and seasonal [26], pandemic [27,28], or both types [29,30,31] of influenza vaccines. The contributions of those studies and this survey offer the opportunity to focus on the need for information and education, in addition to correct perceptions of risk in HCWs and health professions students.

The strengths and limitations of this study are mainly related to its design, as previously described [14,15].

Adapting strategies for the distribution of questionnaires to the specific needs of the HCWs allowed us to obtain information on as many professionals and students as possible. However, this strategy also had the potential to limit the generalizability of the collected data, introducing a confounder. The ability to administer the questionnaire during training courses led to high response rates. In contrast, the use of locked boxes to collect the questionnaires in some departments resulted in lower levels of compliance.

Another limit of the study is represented by the fact that we do not have any information regarding those who did not return the questionnaire; this aspect may further limit the generalizability of the results.

In conclusion, the results highlight the importance of improving vaccination among HCWs through multi-component interventions. Targets should include both HCWs who do not usually get vaccinated or who sometimes get vaccinated and HCWs who have been vaccinated in previous years.

This study also underlines the importance of improving knowledge of influenza transmission and vaccination among HCWs. Moreover, the findings of this study indicate a need to strengthen knowledge of the risks of influenza through university coursework.

## Highlights

- Assessing attitudes of HCWs regarding influenza vaccination can be useful.
- The enrolled subjects tended to respond that they were “never vaccinated” (74.6%).
- Knowledge of influenza and vaccines should be reinforced among those who have never been vaccinated.
- Levels of awareness should be maintained among the “sometimes” and “always vaccinated” subjects.
